# Moving toward a better understanding of renal lymphatics: challenges and opportunities

**DOI:** 10.1007/s00467-025-06692-7

**Published:** 2025-02-03

**Authors:** Jianyong Zhong, Jing Liu, Ashley L. Mutchler, Haichun Yang, Annet Kirabo, Elaine L. Shelton, Valentina Kon

**Affiliations:** 1 Department of Pediatrics, Division of Pediatric Nephrology, Vanderbilt University Medical Center, Medical Center North C-4204, 1161 21st Avenue South, Nashville, TN 37232–2584, USA; 2 Department of Pathology, Vanderbilt University Medical Center, Nashville, TN, USA; 3 Department of Nephrology, School of Medicine, Tongji Hospital, Tongji University, Shanghai, China; 4 Division of Clinical Pharmacology, Department of Medicine, Vanderbilt University Medical Center, Nashville, TN, USA; 5 Vanderbilt Center for Immunobiology, Vanderbilt University Medical Center, Nashville, TN, USA; 6 Vanderbilt Institute for Infection, Immunology and Inflammation, Vanderbilt University Medical Center, Nashville, TN, USA; 7 Vanderbilt Institute for Global Health, Vanderbilt University Medical Center, Nashville, TN, USA

**Keywords:** Kidney, Lymphatics, Interstitial compartment

## Abstract

The development of lymphatic-specific markers has enabled detailed visualization of the lymphatic vascular network that has greatly enhanced our ability to explore this often-overlooked system. Lymphatics remove fluid, solutes, macromolecules, and cells from the interstitium and return them to circulation. The kidneys have lymphatics. As in other organs, the kidney lymphatic vessels are highly sensitive to changes in the local microenvironment. The sensitivity to its milieu may be especially relevant in kidneys because they are central in regulating fluid homeostasis and clearance of metabolites delivered into and eliminated from the renal interstitial compartment. Numerous physiologic conditions and diseases modify the renal interstitial volume, pressure, and composition that can, in turn, influence the growth and function of the renal lymphatics. The impact of the renal microenvironment is further heightened by the fact that kidneys are encapsulated. This review considers the development, structure, and function of the renal lymphatic vessels and explores how factors within the kidney interstitial compartment modify their structure and functionality. Moreover, although currently there are no pharmaceutical agents that specifically target the lymphatic network, we highlight several medications currently used in children with kidney disease and hypertension that have significant but underappreciated effects on lymphatics.

## Introduction

The lymphatic vascular network consists of blind-ended capillaries and unidirectional collecting vessels that coalesce to form a hierarchical network crucial to maintaining fluid, immune, and lipid homeostasis [1, 2]. Lymphatic vessels, found in nearly every organ, transport fluids, solutes, cells, and macromolecules from the interstitial compartments back into the blood circulation. Because of their essential role in regulating body fluid homeostasis and clearance of metabolites, the kidney interstitium is in constant flux [3, 4]. This characteristic of kidney function is important since lymphatic vessels are very sensitive to changes in their local environment. Thus, even minor alterations in interstitial composition and pressure can have amplified effects on renal lymphatic function, which, in turn, can modulate kidney as well as systemic disorders and diseases in distant organs.

This review offers an overview of the development, structure, and function of renal lymphatic vessels. It updates our current understanding of how kidney injury affects lymphatic responses, focusing on how injury-induced factors within the kidney interstitial compartment modify the structure and function of renal and extrarenal lymphatic vessels. Finally, we consider the underappreciated lymphatic effects of several pharmacologic therapies currently used in children with kidney disease and hypertension and discuss the benefits of developing alternative therapeutic strategies.

### Anatomy and architecture of kidney lymphatics

**Fig. 1:**
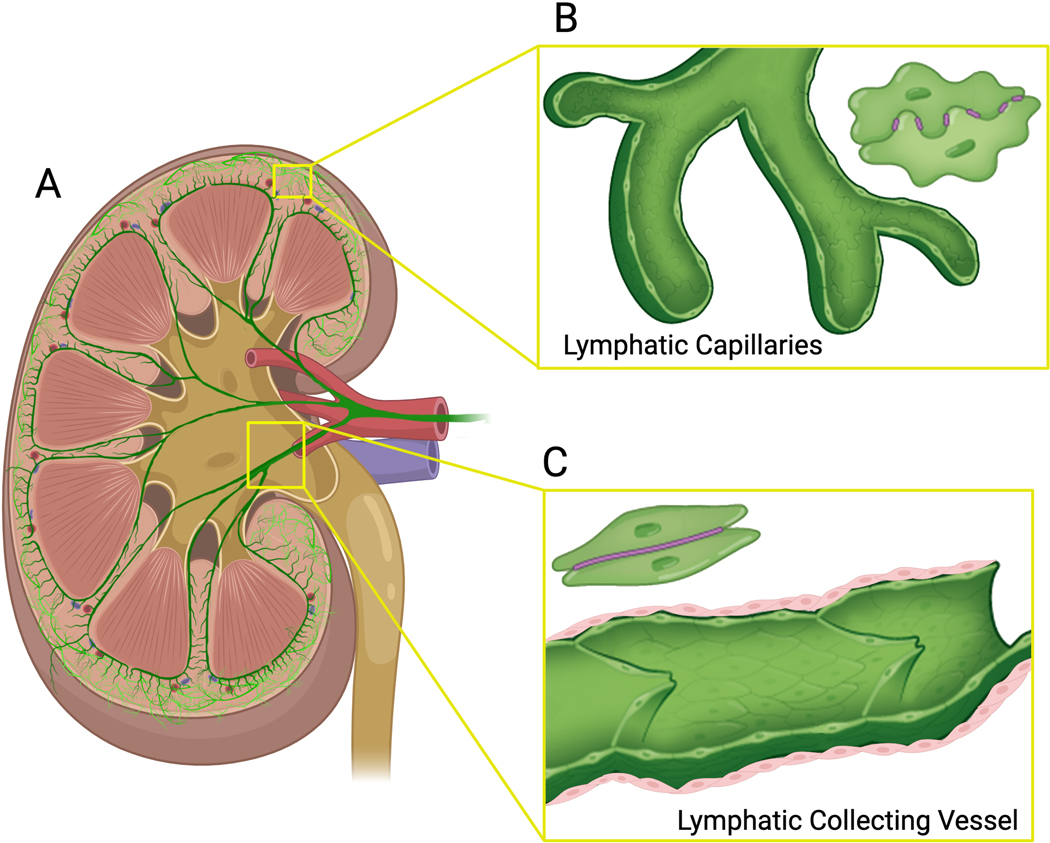
Structure and architecture of the kidney lymphatic network. **A** The lymphatic network begins with blind-ended capillaries in the kidney cortex that coalesce into collecting vessels and follow the arteries and veins toward the hilum. Capsular lymphatics are detected in some species. The renal medulla does not have true lymphatics, but the ascending vasa recta is a hybrid expressing both blood and lymphatic markers. The image is a cross-section of a rat kidney stained with podoplanin, a lymphatic vessel marker that also stains glomerular podocytes. **B** Lymphatic collecting vessels are lined with endothelial cells containing continuous zipper-like cell junctions (purple line between cells), studded with one-way valves within the lymphatic lumen, and ensheathed in smooth muscle (pink cells). **C** The lymphatic capillaries have overlapping endothelial cells with button-like junctions (purple dashed between cells). Images adapted with permission from reference [101] using BioRender Illustration Software

Much of what we know about the basic anatomical layout of renal lymphatics comes from seminal dye perfusion studies performed in a variety of mammalian species [5]. Studies in humans have demonstrated a great deal of structural consistency [4, 6]. The intrarenal/hilar network serves as the primary renal network, originating from blind-ended capillaries that merge and travel in parallel with the corresponding arteries and veins toward the corticomedullary junction. The initial intralobular and interlobular lymphatics combine into arcuate and interlobar pre-collectors and then hilar collectors that drain into lymph nodes [4] (Fig. 1A). Some reports have also described a capsular system [7], but the existence and importance of a secondary network remain somewhat unsettled as more recent studies have not identified this network [6, 8].

A recent study emphasized that the molecular heterogeneity along the kidney lymphatic network mirrors the pattern documented in extrarenal lymphatic beds [9]. The article also demonstrated how injury alters renal lymphatic endothelial cell (LEC) expression of genes linked to VEGF stimulation, VEGF receptor signaling pathways, and lymphatic vessel development. About a quarter of the genes were linked to T-cell differentiation (*Ctla2a, Id1, Cd38, Rsad2*) or regulation of the inflammatory response (*Plpp3, Ifitm3, Bst2*). These changes in the renal environment likely prime LECs for proliferation and modulate lymphatic crosstalk with immune cells.

Similar to other lymphatic beds, renal lymphatic capillaries feature overlapping LECs with button-like junctions and anchoring filaments tethered to the interstitial extracellular matrix (ECM) (Fig. 1B). This configuration prevents vessel collapse and facilitates fluid absorption which allows fluid to flow into the capillary lumen. The initial capillaries merge into pre-collecting and then collecting vessels lined with relatively impermeable LECs containing continuous zipper-like cell junctions and a complete basement membrane, which are wrapped in contractile smooth muscle and studded with intraluminal valves (Fig. 1C) [10]. This arrangement is well-suited to coordinate and propel lymph from the interstitium toward the thoracic duct and into the systemic circulation. As in other organs, the larger valved contractile collecting lymphatic vessels are most abundant at the perimeter, which in the kidney is at the hilum, effectively propelling lymph out of the kidney [11, 12]. In unpublished studies of GFP-conditional mice under Prox1 promoter, we have observed valves in the renal collecting vessels. There are no true lymphatic capillaries in the kidney medulla. However, the medullary ascending vasa recta (AVR) is a hybrid structure containing both blood and lymphatic markers, although the markers have also been observed in cortical peritubular capillaries [13–15]. The AVRs are thought to play a physiological role in the reabsorption of medullary interstitial fluid. Deletion of *TIE2*, a gene critical for AVR development, results in reduced urine concentrating ability and the formation of kidney cysts [13].

While these observations reinforce the idea of an important role of AVRs in urine concentrating ability, the exact function or response to kidney injury is not clear and requires further investigation.

### Development of the renal lymphatic network

Immunohistochemical staining using genetic markers of LECs, including LYVE1, PROX1, and VEGFR3, has furthered our understanding of the development of the kidney lymphatic system. Lee et al. [16] reported formation of the lymphatic plexus in mice starts at the kidney hilum after embryonic day 13 (E13) and noted the appearance of well-defined interlobar and arcuate by E14. In a more recent study, Jafree et al. used several lymphatic markers, optical clearing, and single cell-resolution three-dimensional imaging [17] to identify E14.5 to E18.5 as the critical period for kidney lymphangiogenesis and confirmed that definitive PROX1 + /LYVE1 + lymphatic vessels could not be detected until E14.5. From E14.5 to E15.5, the number of vessels increased slowly, followed by a rapid expansion of the lymphatic plexus until E18.5. This expansion was characterized by a significant increase in lymphatic vessel branch number, length, and diameter of individual vessels and an increase in the volume of the total vascular network. Notably, at E18.5, PROX1 + vessels did not stain for α-SMA, a smooth muscle cell marker, suggesting that pre-collector or collecting vessels had not yet formed. In rats, lymphatic vessels were recognized later in kidney development (E17) and appeared most prominent in the renal hilum and interstitium surrounding the kidney pelvis at E20, although earlier time points were not provided [18]. A small number of lymphatic vessels were observed in the corticomedullary region at postpartum day 1 (P1) and the cortex at P10. Currently, only limited information exists on kidney lymphatic development in humans. Three-dimensional imaging revealed lymphatic vessels in both the hilum and cortex are present by post-conception week 12, a time point that corresponds to the accelerated expansion observed in mice at E15.5 [17, 19].

Interestingly, the timing of lymphatic vessel development corresponds to dramatic changes occurring in other parts of the kidney. At E14.5, the mesonephros begins to degenerate, while glomeruli, stem cells, and abundant blood vessels become readily detected, and the ureteric buds undergo 8–9th generations of branching [20]. The rapid development of kidney lymphatics at E15.5 suggests that these developmental changes may reflect mechanical stimulation from the accumulation of interstitial fluid, although this possibility remains to be proven [21, 22]. This is also the time when kidney excretory function begins, a process that predicts a reduction in interstitial congestion [21]. Kidney lymphatic development also coincides with the upregulation of key paracrine factors, namely, vascular endothelial growth factor C (VEGF-C), observed within the developing renal arteries [17]. Overall, while lymphatic growth parallels kidney development, further studies are needed to determine how lymphatic reabsorption and clearance of the interstitial compartment influence this developmental and maturational process.

### Kidney lymphatics and disease modulation of the lymphatic network

The lymphatic network regulates tissue fluid balance by clearing the interstitial compartment. The magnitude of this function is striking. Witte et al. measured the thoracic duct lymph flow which represents the total volume of fluid cleared from all tissues [23]. In healthy subjects, the thoracic volume of 1.4 L/day is an amount remarkably similar to the urinary flow rate. In patients with severe congestive heart failure, the volume was > 11 L/day, observations that underscore the importance of the lymphatic pathways in clearing interstitial fluid in edema-forming conditions.

**Fig. 2:**
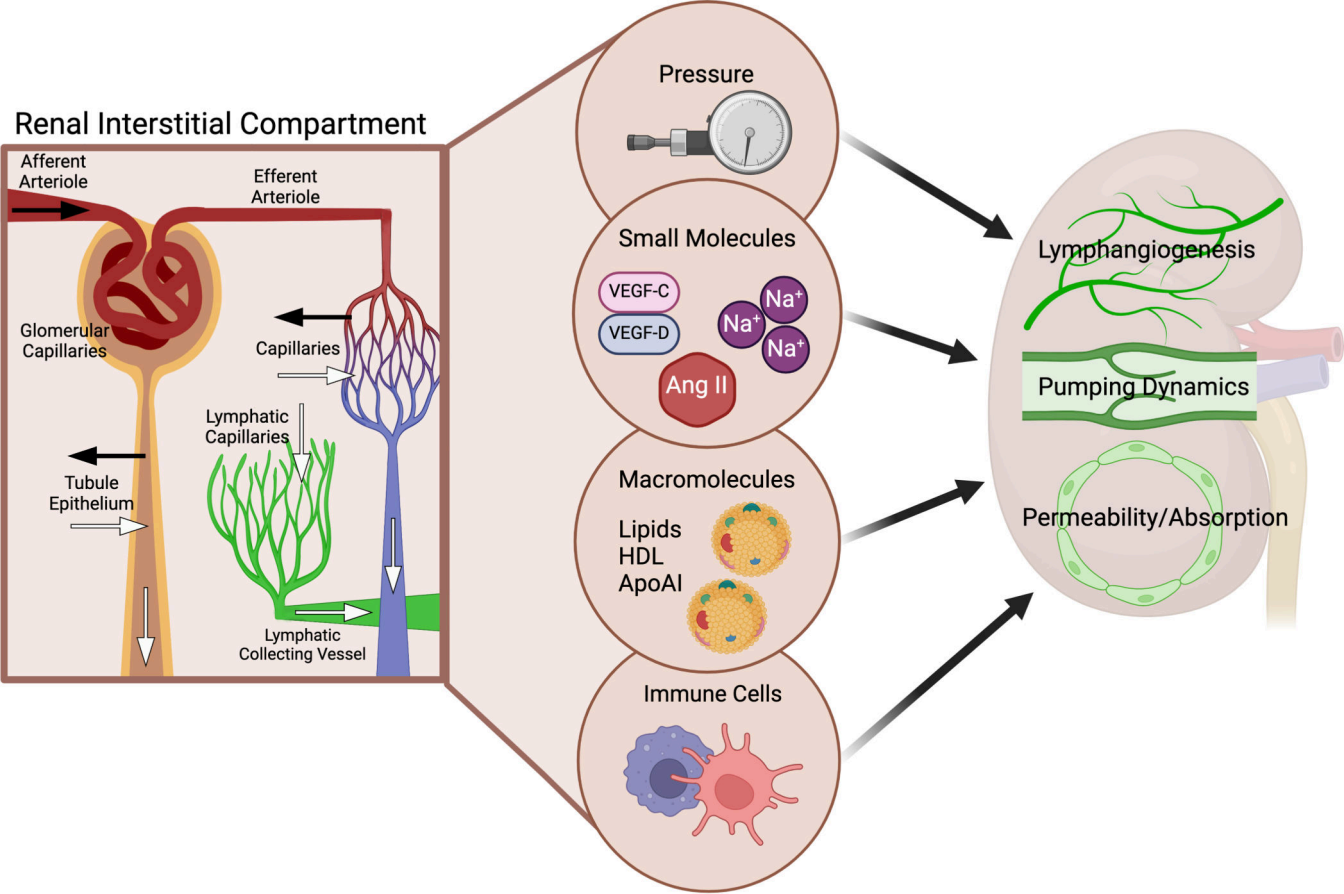
Factors in kidney interstitium influence lymphatic vascular network. The interstitial compartment in the kidney receives fluid and solutes from arterioles/capillaries and tubular reabsorption (black arrows), while the outflow occurs through veins, lymphatics, and tubular excretion (white arrows). Physiologic and pathophysiologic factors can affect the volume and composition of the interstitial compartment, including hydraulic pressure, solutes and small molecules (such as sodium, vasoactive, and growth factors), macromolecules (such as lipids), and immune cells. These factors, both individually and cumulatively, regulate lymphangiogenesis, pumping dynamics, and the permeability/absorption capacity of the kidney lymphatics

Although kidney lymph is a relatively small contributor to the total thoracic volume [4], the kidney lymphatic vessels profoundly affect total body fluid balance because of the kidneys’ unique role in fluid homeostasis and elimination of various metabolites. The kidney interstitium is in constant flux, and the renal lymphatic vessels are essential in clearing interstitial fluid, solutes, macromolecules, and infiltrating immune cells. Unlike the interstitial compartment in other organs where arterioles bring in blood and veins and lymphatics drain it, the inflow into the kidney interstitial compartment is also supplied by reabsorption from the tubules. Unlike other organs, fluids, tubule secretion, and urinary excretion drain the renal interstitium. The anatomical organization is particularly important because the kidneys are encapsulated, and the accumulation of fluid and biologically active molecules can directly affect the lymphatic vessels, which are exquisitely sensitive to the local environment. An overview of these mediators is illustrated in Fig. 2.

Kidney lymphatics respond to physiologic and injurious stimuli. The most extensively documented phenotypic change is lymphangiogenesis which has been recognized in numerous clinical experiments and in acute kidney injury (AKI), chronic kidney disease (CKD), kidney transplants, and hypertension [8, 24–26]. Recent reports suggest that the extent of lymphatic expansion seen in kidney biopsies may predict poor prognosis [27]. Elevated serum and urine levels of lymphangiogenic markers, LYVE1 and VEGF-C, in serum and urine, have also been linked to increased kidney fibrosis [28]. The lymphangiogenic response reflects increased secretion of VEGF-C and VEGF-D by kidney tubules and infiltrating macrophages [29, 30]. While injury and disease stimulate lymphatic growth, it remains unclear whether lymphangiogenesis is beneficial or harmful. A potential beneficial effect is the clearance of fluid, immune cells, pro-inflammatory cytokines, and cellular debris. However, the formation of new lymphatic vessels is frequently disorganized, resulting in leakage and further exacerbating the inflammatory injury [31]. Indeed, kidney injury affects lymphatic vessel dynamics.

Less studied than lymphangiogenesis, lymphatic contractility is increasingly recognized as an important contributor to tissue clearance. It is a technically challenging assessment. The contractility and transport by lymphatics have been analyzed using a variety of in vivo and ex vivo techniques (reviewed by Zawieja et al. [32]). The earliest in vivo studies were primarily descriptive and employed intravital microscopy in rats and guinea pigs to visualize contractions of testicular, mesenteric, and inguinal collecting vessels [33, 34]. Advances in the ability to record in microvessel diameter changes and fast video microscopy techniques facilitated evaluations of pressure and lymph flow via particle velocity tracking [35]. More recently, vessel contractility was analyzed by injecting a fluorescent dye enabling visualization and assessment of collecting vessel diameter as a function of time [36]. While useful, these in vivo techniques require lymphatic vessels to be surrounded by thin relatively transparent tissues, like the mesentery, for visualization. This limits which vessels can be studied. Additional constraints relate to the use of anesthesia to immobilize study animals which minimizes the contribution of extrinsic factors (respiration, skeletal muscle contraction, intestinal motility) on lymphatic transport and direct toxic and pressure altering effects of some dyes [37, 38].

Ex vivo analysis methods mitigate some of the disadvantages of in vivo approaches in that they allow for vessels from a wider variety of source tissues to be studied. Also, vessels can be analyzed without the potentially confounding influences of neural or immune components on vasoreactivity. Early ex vivo studies using large bovine mesenteric lymphatics demonstrated that isolated collecting vessels retained their ability to contract spontaneously even in the absence of innervation and native tethering forces [39]. These studies foreshadowed the use of organ bath myography systems to study lymphatic contractility and valve dynamics in smaller caliber vessels. Specifically, pressure myography assays, which more closely mimic native physiologic conditions, have been useful to study pumping dynamics [40] and lymphatic valve competency [41, 42] in response to pharmacological factors and biomechanical stimuli.

Recently, we showed that rats with proteinuric injury have dilated afferent renal collecting vessels, decreased magnitude of contraction, and diminished ejection fraction compared to vessels from healthy rats [43]. These lymphatic changes predict reduced clearance of the kidney interstitium and further disarray within the interstitial environment. Figure 2 highlights specific factors within the kidney interstitial environment linked to changes in the lymphatic phenotype.

### Pressure

As noted above, the accumulation of interstitial edema indicates a failure of compensatory mechanisms [44]. Wilcox et al. documented elevated interstitial pressure in rats with lymphatic-ligated kidneys [45]. The elevation in interstitial pressure was further amplified following volume expansion. Other studies confirm that physiological and pathological conditions, such as saline expansion, natriuresis, venous hypertension, and elevated systemic blood pressure, alter kidney interstitial pressure [46–51]. Increased intrarenal interstitial pressure, whether due to excess inflow (such as volume overload) or impaired urinary, venous, or lymphatic outflow (obstruction), can affect lymphatic vessel growth, reabsorption capacity, contractility, and lymph flow.

Elevated kidney interstitial pressure can even cause the collapse of intrarenal collecting lymphatics, further disrupting lymph flow [4]. These scenarios suggest that beyond a certain level of interstitial edema, lymph flow will not increase, resulting in further edema and deterioration of kidney function. Indeed, long-term studies of lymphatic ligation and edema within the kidney have reported fibrosis in animal models [52].

Interventions increasing kidney lymphatics that encourage interstitial clearance and lower pressure have also been done. For example, conditional kidney-specific VEGF-D overexpressing mice or targeted delivery of micellar nanoparticles loaded with a VEGFR-3-specific mutant of VEGF-C (VEGF-C c156s) or free VEGF-C protein expanded the kidney lymphatic network, promoted sodium excretion, and lowered systemic blood pressure in several hypertensive mouse models [53–55]. These interventions predict a reduction in the interstitial pressure; however, direct pressure measurements were not done. Indeed, it should be underscored that while there is ample evidence that various physiological stimuli and disease conditions influence renal interstitial pressure, lymphangiogenesis, and vessel dynamics [3, 4, 53, 55, 56], there are no studies that show a direct cause-and-effect relationship between altered renal interstitial pressure and renal lymphatic dysfunctions.

### Solutes and small molecules

High-sodium environments significantly impact lymphatic vessels. Much of the sodium-induced lymphangiogenesis research has focused on salt-sensitive hypertension linked to elevated sodium levels in the skin [57]. More recent studies have found sodium accumulation, along with water, is not confined to the skin and muscle but appears to be systemic, affecting other organs, including the lung, liver, and heart [58]. Using 23Na-MRI, significant sodium and water accumulation was observed in the kidneys of puromycin-injured rats [43]. This study also reported that sodium concentration in lymph from the proteinuric kidneys was significantly higher than in lymph from uninjured control rats, despite no difference in blood sodium levels between the two groups. These results echo earlier studies that observed renal lymph/plasma sodium ratios ranged between 1.03 and 1.07 [4, 59, 60]. Similarly, increased sodium concentration was also documented in lymph collected from dermal lymphatic vessels of salt-sensitive hypertensive rats [61]. Together, these observations highlight that lymph composition reflects the interstitial environment of the draining organ. Direct evidence that sodium regulates lymphatic dynamics was shown by ex vivo studies using isolated vessel myography. Renal lymphatic vessels exposed to a high-sodium environment reduced contractility that reflected increased end-systolic diameter, reduced contraction amplitude, and ejection fraction that involved the SPAK-NKCC1-eNOS pathway [43]. Similar lymphatic dynamics were observed in rat skin and muscle lymphatic vessels exposed to a high salt diet or deoxycorticosterone acetate (DOCA) [62].

Aside from sodium and water, renal lymphatics appear critical in the clearance of other molecules with relevance to kidney function and kidney disease. Thus, the renin–angiotensin–aldosterone system (RAAS) is pivotal in the physiologic regulation of tubular reabsorption of salt and water, in modulating arteriolar tone and ultrafiltration capacity of the glomerular capillary bed, and activation of profibrotic pathways. Remarkably, angiotensin II (AngII) levels in kidney lymph are 10–100 × higher than in kidney veins or in the circulating plasma consistent with enrichment of Ang II within the renal interstitial compartment [63–66]. The original observations connected the renal lymphatic enrichment in AngII to renin release by the juxtaglomerular cells [66]. The novel implication of these old findings is that the renal lymphatic vessels participate in the clearance of Ang II from the renal interstitium. Thus, in the setting of kidney injury, disruption in lymphatic dynamics may promote intrarenal accumulation of Ang II that amplifies its renal actions. An additional implication is that beyond inhibition of the RAAS, enhanced lymphatic elimination of intrarenal AngII is an attractive therapeutic target. Currently, little is known about how lymphatic dysfunction affects AngII clearance.

### Large molecules

One of the critical functions of lymphatic vessels is the absorption and transport of dietary lipids from the intestines to the circulation [67]. Lymphatics also mobilize and remove excess tissue cholesterol, incorporating it into lipoprotein acceptors like apolipoprotein AI (apoAI) and forming high-density lipoprotein (HDL), which is transported to the liver for excretion in bile [68]. Kidneys are increasingly recognized as participants in lipid metabolism, and kidney diseases can lead to renal lipid accumulation [69]. Nonetheless, the extent to which kidney lymphatics affect lipid handling or accumulation is not fully understood. Our research has demonstrated that kidney lymphatics transport apoAI, including apoAI modified by the peroxidation product, IsoLG, which activates lymphatic endothelial cells [29]. These findings contribute to the growing literature indicating that kidneys play a critical role in the kidney metabolism of large molecules, although the role of lymphatic vessels requires further investigation.

### Immune cells

Another vital function of the lymphatic system is immune surveillance and the adaptive immune response. Kidney disease increases immune cell infiltration into the kidneys, and lymphatic vessels play a crucial role in clearing these cells from the interstitial compartment. The migration, intravasation, and intraluminal crawling of immune cells through the lymphatic vessel walls involve direct interaction with LECs [70]. Activated CD11c + dendritic cells (DCs) engage in direct interactions with LECs, thereby modulating lymphatic vessel function and LEC proliferation. Classically, activated M1 macrophages can transdifferentiate into LECs through VEGF-C/VEGFR3 activation in kidney fibrosis [71]. In studies submitted for publication, we recently showed that a high-sodium environment following nephrotoxic kidney injury in mice activates crosstalk between infiltrating immune cells and LECs. The immune cell-LEC crosstalk involves the vasoactive endothelin 3 generated by the immune cells and its receptor, endothelin B receptor, on LECs. In cultured cells, the ET3:ETBR axis promoted immune cell migration and increased attachment to LECs. In isolated lymphatic vessels, high ET3 levels minimized contractility that could amplify stagnation of the activated immune cells and advance inflammation and progressive kidney damage.

### Extrarenal lymphatics

Kidney disease not only affects lymphatic vessels within the kidney but also influences extrarenal lymphatics. Proteinuric kidney injury [72] significantly expanded the intestinal lymphatics, increased lymph flow, and altered the pumping dynamics in the mesenteric bed. In animals with kidney injury, isolated mesenteric lymphatic vessels showed increased contraction frequency but a marked reduction in contraction amplitude and end-diastolic diameter compared to healthy animals. It is notable that kidney disease alters the intestinal microenvironment in much the same way as the renal microenvironment including increased sodium, immune cells, and altered microbiome that directly modulate the intestinal lymphatic network [73]. Kidney injury also affected the composition of mesenteric lymph, increasing the output of cholesterol, triglycerides, apoAI, and lipid peroxides, particularly the highly reactive IsoLG. Mesenteric lymph from kidney-injured animals also showed increased levels of Th17 cells and cytokines such as IL-6, IL-10, and IL-17. These observations underscore that in addition to the well-substantiated intestinal damage (such as disruption of the intestinal epithelial barrier, the microbiome, generation of toxins), kidney disease disorders lymphatics in distant organs although the precise mechanism remains elusive.

### The relationship of abnormal lymphatics to kidney development or disease

Genetic mutations in *VEGFR3, PROX1, FOXC2, PIEZO*, and *SOX18* are known to cause specific diseases [74, 75]. For example, autosomal-dominant loss-of-function *FLT4* gene mutations underlie Nonne-Milroy lymphedema of the lower limbs, while homozygous and compound heterozygous mutations in the mechanically activated ion channel PIEZO1 result in an autosomal recessive form of dysplasia with a high incidence of non-immune hydrops fetalis and childhood facial and limb lymphoedema [76, 77]. Little is known about the role of lymphatic-related proteins in kidney development. Recently, a mouse model containing a missense mutation in *Vegfr3* (named *Chy*) that abolishes its kinase capacity examined the impact of the mutation on kidney development and function. Homozygous Vegfr3Chy/ + mice do not survive; however, heterozygous Vegfr3Chy/ + mice had about half the total lymphatic volume compared to control mice at 12 weeks of age [78]. Despite the reduced quantity of lymphatic vessels, the mice showed minimal difference in kidney structure or function compared to controls or after mild low-dose cisplatin injury. The authors suggested that while the reduced lymphatic density appears adequate under normal or mild injury conditions, it may be insufficient during more serious kidney stress, such as chronic salt loading or severe injuries.

Recent research has proposed that abnormal lymphatics may play a causative role in cyst development in polycystic kidney disease (PKD), characterized by fluid-filled epithelial cysts in the kidneys that lead to inflammation and fibrosis [79]. Traditionally, localization of *Pkd1/2* in tubular epithelial cells has been considered the common cause of polycystic kidney disease. Localization of *Pkd1/2* to LECs has triggered the exploration of lymphatic vessel-related polycystic kidney disease [80]. *Pkd1* regulates LEC migration and morphogenesis in zebrafish and mice. *Pkd1* endothelial cell-specific KO mice exhibit lymphatic malformations, including a reduced proportion of lymphatic vessels relative to kidney mass and decreased lymphatic branching [80, 81]. The lymphatic deficiency appears before the development of kidney cysts, indicating that *Pkd1* mutations directly disrupt lymphatic development through its role in LECs rather than indirectly through edema in polycystic kidneys.

Impaired clearance of the kidney interstitial compartment has been linked to systemic hypertension and kidney pathology [53], although the specific impact of delayed/ineffective lymphatic clearance during kidney development is not fully understood. Lilienfeld et al. reported that ligating hilar and capsular lymphatics in dog kidneys, which likely increased intrarenal interstitial pressure, caused a prompt 20 mmHg rise in systemic blood pressure [82]. Conversely, more recent studies found that elevated systemic blood pressure can be reduced in several hypertensive mouse models in association with expansion of the kidney lymphatic network by kidney-specific overexpression of VEGF-D or targeted kidney delivery of VEGF-C [54–56, 83]. Although the kidney interstitial pressure was not measured in these studies, the authors concluded that expanding the kidney lymphatic network promotes lymph flow and urinary excretion, thereby reducing interstitial congestion and lowering systemic blood pressure.

### Lymphatics as therapeutic targets

#### Strategies targeting lymphatic vessels

As discussed above, numerous animal models and clinical studies have demonstrated that kidney injury leads to kidney lymphangiogenesis. While this association is well-established, whether the lymphatic expansion is a cause or consequence and whether it is beneficial or detrimental is unclear and likely reflects the type and stage of the disease. This ambiguity may influence the effectiveness of therapeutic interventions. Currently, most pharmacologic approaches focus on targeting lymphatic vessel growth. Given that VEGF-C/VEGFR3 is a critical axis in lymphangiogenesis, much attention has been given to targeting components of this pathway [84–86]. Therapies using VEGF-C modulators also affect blood vessels, as VEGF-C binds to VEGFR-2 in addition to VEGFR-3 [87]. This unintentional interaction highlights potential vascular side effects of lymphangiogenic therapies on arteries and veins, including angiogenesis or capillary leak that may have beneficial or harmful consequences. This issue was highlighted in a recent study that used a mouse model expressing Vegf-C under the regulation of the nephron progenitor Six2Cre driver strain (Six2Vegf-C) [88]. The gain-of-function model of tubule overexpression of VEGF-C increased renal lymphangiogenesis but showed little effect on the growth of blood capillaries. Critically, however, the mice developed a severe cystic phenotype and renal insufficiency. Although this study used a constitutive knockout that may obscure the acute effects of VEGF-C therapy and did not specifically address a VEGF-C-VEGFR2 pathway, the off-target consequences of increasing VEGF-C were striking. These results underscore the need for new strategies in drug delivery systems that preferentially target lymphatics and/or biomaterials specifically designed to home in lymphatic vessels. Currently, no drugs specifically target kidney lymphatic vessels in clinical practice.

### Underappreciated lymphatic effects of currently used therapies

Contrasting the efforts to modulate lymphatic growth, pharmacological research has largely overlooked the impact of drugs on lymphatic contractility and lymph flow. As noted, in large part, this omission reflects difficulties in visualizing lymphatics and lymph flow in vivo and the technical challenges in identifying, retrieving, and measuring dynamics in isolated lymphatic vessels ex vivo. Despite these limitations, a number of FDA-approved drugs are now recognized to influence lymphatic contractility. Russell et al. compiled a comprehensive list of clinically relevant drugs with known effects on lymphatic contractile functions [89]. Table 1 lists drugs likely prescribed to pediatric nephrology patients that affect lymphatic contractility. Here, we focus on several commonly used therapeutics, specifically medications prescribed to treat hypertension and volume overload and to induce remission of nephrotic syndrome.

#### Antihypertensives

The review by Russell et al. of over 200 drugs affecting lymphatic vessels highlighted that most drugs diminish lymphatic contractility [89]. Mechanistically, lymphatic contractility is regulated by a cascade that includes intracellular calcium accumulation, which involves membrane depolarization-based activation of L-type voltage-dependent calcium channels and IP3-mediated release of calcium from sarcoplasmic stores [90]. Thus, ion channels and transporters play significant roles in lymphatic membrane potential, and many commonly used medications fall into this category. Among these, calcium channel blockers (CCBs) are the most frequently used drugs that inhibit lymphatic pumping [89].

Calcium channel blockers, including verapamil, amlodipine, and diltiazem, are often used to treat hypertension in children. These CCBs are well known to cause peripheral edema that has been attributed to arteriolar dilatation and fluid leakage from blood capillaries into the interstitial space [91]. This scenario should be updated to include clearance of the interstitial compartment by lymphatic vessels. CCBs reduce the lymphatic tone and phasic contraction, leading to decreased interstitial clearance and, thus, edema formation [89]. Interestingly, individuals with low baseline lymphatic contractility are more likely to develop edema when treated with CCBs [92]. Since kidney disease impairs the contractility of kidney-collecting vessels [43], CKD patients might be particularly susceptible to CCB-induced edema. In this connection, a recent report described patients undergoing peritoneal dialysis who developed cloudy peritoneal dialysate shortly after starting CCBs [93]. The dialysate cloudiness was not due to infection and the peritoneal fluid cleared within 24–72 h of withdrawal of the drug. Remarkably, the re-administration of CCBs led to the recurrence of chyloperitoneum in several individuals. Chyloperitoneum secondary to the use of CCBs has also been reported in the general population [94]. Notably, polymorphisms in a calcium channel gene have been linked to a higher susceptibility to chylous ascites [95].

Though less commonly prescribed than CCBs, ATP-gated potassium (KATP) channel openers like pinacidil and diazoxide are also used for treating hypertension and also cause peripheral edema that may reflect hyperpolarization in lymphatic muscle cells and reduced lymphatic contractility and lymph flow [96]. The FDA-approved KATP antagonist glibenclamide rescued lymphatic contractile dysfunction in a mouse model with gain-of-function mutations in the *Kir6.1* gene, which mimics lymphoedema seen in patients with Cantú syndrome. Glibenclamide also improved mesenteric lymphatic contractility in a metabolic syndrome model [32]. This drug represents one of the few pharmaceutical agents that enhances lymphatic contractility and thus may offer new therapeutic avenues.

#### Diuretics

Diuretics are the first-line therapy to treat sodium overload in various diseases, including hypertension and kidney disease in children. Furosemide, the most frequently prescribed diuretic, inhibits NKCC1 and NKCC2, two isoforms of the Na–K–Cl (NKCC) cotransporter. The NKCC2 isoform facilitates ion transport into epithelial cells of the thick ascending limb of the loop of Henle, and NKCC2 inhibition by furosemide enhances kidney excretion of both water and solutes. The NKCC1 isoform modulates tone in blood vessels by increasing Cl − efflux, which depolarizes the membrane and increases cellular Ca + + levels, thus enhancing contractility [97]. We have shown that Nkcc1, but not Nkcc2, is also expressed in kidney lymphatic collecting vessels [40]. Our functional studies revealed that direct exposure of isolated kidney-collecting vessels to furosemide decreases the frequency of contractions and reduces the magnitude of contractions and ejection fraction that could diminish clearance of the interstitial compartment and, therefore, counteract the tubular excretion of sodium and water. Interestingly, we found that another loop diuretic, ethacrynic acid, had minimal effect on the kidney vessels. The discrepant effects may be due to earlier observations that furosemide, but not ethacrynic acid, affects calcium release from the sarcoplasmic reticulum of vascular smooth muscle cells [98]. Since kidney injury increases interstitial sodium and elevates interstitial pressure, each of which impairs lymphatic contractility [43], furosemide inhibition of NKCC1 may further exacerbate lymphatic dilation and reduce contractility, which would reduce tissue clearance within the kidney and peripheral tissue. This scenario may explain why some patients with kidney disease develop resistance to diuretic therapies.

#### Anti‑inflammatory/immunosuppressives

Corticosteroid treatment constitutes the therapeutic cornerstone in proteinuric nephropathies in children. While the immunomodulatory actions effectively induce clinical remission, steroids acting through glucocorticoid receptors inhibit VEGF-C-induced lymphangiogenesis [99]. Besides blunting lymphatic growth, corticosteroids promote the transition of the zipper-to-button junctions that facilitate the clearance of tissue inflammatory cells and cytokines involved in resolving inflammation [99]. Recently, we found that dexamethasone applied directly to isolated kidney-collecting lymphatic vessels reduces contraction amplitude and ejection fraction (submitted for publication). Since lymphatic vessels have a key role in adipocyte growth, lipid absorption, and transport, steroid-induced lymphatic dysfunction in lymphatic contractility may be a contributing mechanism to visceral adiposity, a common consequence of long-term steroid therapy. Nonsteroidal anti-inflammatory drugs (NSAIDs) also modulate lymphatic vessels, with their impact depending on inflammation levels. During inflammation, NSAIDs increase lymphatic contractility [100].

## Summary/conclusions

This review underscores several findings: (1) Lymphatic vessel development parallels the maturation of the kidneys, suggesting that lymphatic clearance of the interstitial compartment may be a crucial step in kidney maturation. Additionally, abnormal lymphatics are associated with developmental abnormalities of the kidneys. (2) Components within the kidney interstitium, e.g., pressure, sodium, vasoactive substances, lipids, and immune cells, can modulate lymphatic vessel function. (3) While much of the advancements in lymphatic biology and therapy have focused on the growth of lymphatic vessels, additional focus should be given to assessments of vessel contractility to provide a more holistic understanding of lymphatic vessels. We suggest that at least some of the benefits of lymphangiogenesis depend on the functionality of these new vessels. (4) While no specific drugs target lymphatic contractility, there is increased recognition that several commonly used drugs have underappreciated dampening effects on lymphatic pump functions. Identifying these drugs is important for clinicians to choose alternatives with fewer lymphatic effects. Moreover, identifying factors that specifically improve lymphatic function could positively impact kidney disease management.

## Supplementary Material

Graphical Abstract

Supplementary Information

The online version contains supplementary material available at https://doi.org/10.1007/s00467-025-06692-7.

## Figures and Tables

**Table 1.** Clinical drugs with underappreciated contractile lymphatic effects in pediatric nephrology

Therapeutic class	Drug	Lymphatic effect	Ref
**Antihypertensives**	L-type Calcium channel blocker (nifedipine, verapamil, diltiazem)	Lymphatic contraction frequency, contraction amplitude ↓	[89, 93–95, 103–105]
	K_ATP_ agonist (pinacidil)	Lymphatic vessel tone, contraction frequency, contraction amplitude ↓	[40, 106–108]
	α-Adrenergic receptor antagonist (clonidine)	Lymphatic vessel tone ↓	[109–111]
	β-Adrenergic receptor antagonist (propranolol, labetalol)	Lymphatic contraction frequency, contraction amplitude ↓	[112–114]
**Diuretics**	NKCC inhibitors (furosemide, bumetanide) Notably, another NKCC inhibitor, ethacrynic acid, did not affect lymphatic dynamics)	Lymphatic vessel tone, contraction frequency, contraction amplitude ↓	[40, 89, 115]
**Anti-inflammatory/ immunosuppressives**	Corticosteroids (via glucocorticoid receptor: dexamethasone, prednisolone and via glucocorticoid receptor /mineralocorticoid receptors: hydrocortisone)	Depends on duration, dose, inflammatory status. Glucocorticoids improve acute LPS-reduced tone, frequency of contraction. Chronic exposure ↓ contraction amplitude and ejection fraction Mineralocorticoids ↑ contraction frequency, amplitude at lower doses	[116, 117]
	Nonsteroidal anti-inflammatory drugs (NSAIDS) via cyclooxygenases enzymes (ibuprofen, celecoxib, niflumic acid)	Inhibitory/stimulatory lymphatic effects depend on inflammatory status with contractile effects ↑ in inflammatory conditions	[101, 118]

## Abbreviations

AKI: Acute kidney injury
AngII: Angiotensin II
apoAI: Apolipoprotein AI
AVR: Ascending vasa recta
Bst2: Bone marrow stromal cell antigen 2
CCB: Calcium channel blocker
Cd38: Cluster of differentiation 38
CKD: Chronic kidney disease
Ctla2a: Cytotoxic T lymphocyte-associated protein 2 alpha
DC: Dendritic cell
DOCA: Deoxycorticosterone acetate
ECM: Extracellular matrix
eNOS: E ndothelial nitric oxide synthase
ET3: Endothelin-3
ETBR: E ndothelin type B receptor
FLT4: Fms-like Tyrosine Kinase 4
FOXC2: Forkhead Box Protein C2
GFP: Green fluorescent protein
HDL: High-density lipoprotein
Id1: Inhibitor of differentiation 1
Ifitm3: Interferon-induced transmembrane protein 3
IL-6: Interleukin-6
IL-10: Interleukin-10
IL-17: Interleukin-17
KATP: ATP-gated potassium channel
Kir6.1 (KCNJ8): Potassium inwardly rectifying channel subfamily J member 8
KO: Knockout
LEC: Lymphatic endothelial cell
LYVE1: Lymphatic vessel endothelial hyaluronan receptor 1
NKCC: Na-K-Cl cotransporter
NSAID: Nonsteroidal anti-inflammatory drug
PKD: Polycystic kidney disease
Plpp3: Phospholipid phosphatase 3
PROX1: Prospero-related homeobox transcription factor 1
RAAS: Renin-angiotensin-aldosterone system
Rsad2: Radical S-adenosyl methionine domain containing 2
SOX18: SRY-Box transcription factor 18
SPAK: STE20/SPS1-related proline/alanine-rich kinase
TIE2: Tyrosine kinase with immunoglobulin like and EGF like domains 1
VEGF-C: Vascular endothelial growth factor C
VEGF-D: Vascular endothelial growth factor D
VEGFR3: Vascular endothelial growth factor receptor 3
