# Supplementary material for: Moving toward a better understanding of renal lymphatics: challenges and opportunities
Source: Pediatr Nephrol. Author manuscript; Available in PMC 2025 Nov 12. (PMC12402047; doi:10.1007/s00467-025-06692-7)
Supplement: Graphical Abstract [file NIHMS2062884-supplement-Graphical_Abstract.pdf]

Moving Towards a Better Understanding of Renal Lymphatics: Challenges and Opportunities

Review

Key points:

1. Lymphatic vessel development parallels kidney maturation. Abnormal lymphatics are associated with developmental abnormalities of the kidneys.

2. Factors within the kidney interstitium modulate lymphatic vessels.

3. Advancement in lymphatic biology and therapy have focused on lymphangiogenesis, we consider vascular dynamics to further our understanding of normal and pathologic role of lymphatic vessels.

4. Although no specific drugs target lymphatic contractility, some commonly used drugs have underappreciated effects on lymphatic pump functions.

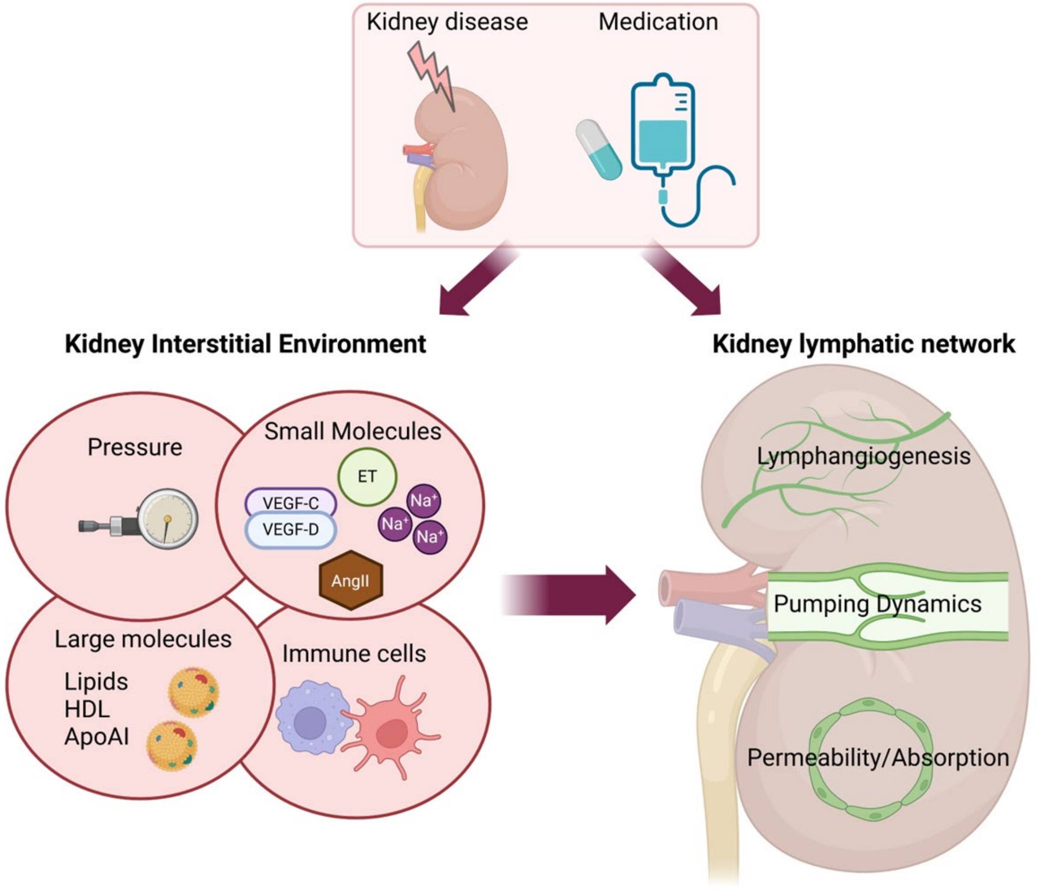

The review considers development, structure, and function of renal lymphatic vessels and explores how disease modifies constituents within the kidney interstitium which modulate lymphatic architecture and actions. Currently, no medication specifically targets lymphatics, but some drugs in clinical use have underappreciated lymphatic effects.

[Zhong and Liu] et al. 2024

IPNA Pediatric Nephrology
Journal of the
International Pediatric Nephrology Association
